# Long-Term Follow-Up and Optimization of Interleukin-1 Inhibitors in the Management of Monogenic Autoinflammatory Diseases: Real-Life Data from the JIR Cohort

**DOI:** 10.3389/fphar.2020.568865

**Published:** 2021-01-11

**Authors:** Véronique Hentgen, Isabelle Koné-Paut, Alexandre Belot, Caroline Galeotti, Gilles Grateau, Aurelia Carbasse, Anne Pagnier, Pascal Pillet, Marc Delord, Michael Hofer, Sophie Georgin-Lavialle

**Affiliations:** ^1^ Department of Pediatrics, National Reference Center for Auto-inflammatory Diseases and Amyloidosis, CEREMAIA, Versailles Hospital, Versailles, France; ^2^ Department of Pediatric Rheumatology National Reference Center for Auto-Inflammatory Diseases and Amyloidosis, CEREMAIA, CHU du Kremlin Bicêtre, University of Paris Sud Saclay, UVSQ, Le Kremlin Bicêtre, France; ^3^ Pediatric Nephrology, Rheumatology, Dermatology, HFME, Hospices Civils de Lyon, National Referee Centre RAISE, & INSERM U1111, Université de Lyon, Lyon, France; ^4^ Department of Internal Medicine, National Reference Center for Auto-Inflammatory Diseases and Amyloidosis, CEREMAIA, Tenon Hospital, AP-HP, Sorbonne University, Paris, France; ^5^ Department of Pediatrics, Hôpital Arnaud de Villeneuve, CHRU Montpellier, Montpellier, France; ^6^ Department of Pediatrics, CHU de Grenoble, Grenoble, France; ^7^ Department of Pediatrics, Hôpital des Enfants, CHRU Bordeaux, Bordeaux, France; ^8^ Direction de La Recherche Clinique et de L’Innovation (DRCI) Versailles Hospital, Versailles, France; ^9^ Unité Romande D’Immuno-Rhumatologie Pédiatrique, CHUV, University of Lausanne, Lausanne, Switzerland

**Keywords:** anakinra, canakinumab, cryopyrin-associated periodic syndrome, Tumor Necrosis factor (TNF)-receptor-associated periodic syndrome, mevalonate kinase deficiency, posology, familial mediterranean fever disease, IL-1 inhibitor

## Abstract

**Objectives:** The major role of interleukin (IL)-1 in the pathogenesis of hereditary recurrent fever syndromes favored the employment of targeted therapies modulating IL-1 signaling. However the best use of IL1 inhibitors in terms of dosage is difficult to define at present.

**Methods:** In order to better understand the use of IL1 inhibitors in a real-life setting, our study assessed the dosage regimens of French patients with one of the four main hereditary recurrent fever syndromes (Familial Mediterranean Fever (FMF), TNF receptor associated periodic syndrome (TRAPS), cryopyrin associated periodic fever (CAPS) and mevalonate kinase deficiency). The patients were retrieved retrospectively from the JIR cohort, an international platform gathering data of patients with pediatric inflammatory diseases.

**Results:** Forty five patients of the JIR cohort with a hereditary recurrent fever syndrome had received at least once an IL1 inhibitor (anakinra or canakinumab). Of these, 43% received a lower dosage than the one suggested in the product recommendations, regardless of the type of the IL1 inhibitor. Especially patients with FMF and TRAPS seemed to need lower treatment regimens; in our cohort none of the FMF or TRAPS patients received an intensified dose of IL-inhibitor. On-demand treatment with a short half-life IL-1 inhibitor has also been used successfully for some patients with one of these two conditions The standard dose was given to 42% of the patients; whereas an intensified dose of IL-1 inhibitors was given to 15% of the patients (44% of CAPS patients and 17% of mevalonate kinase deficiency patients). In our cohort each individual patient’s need for treatment seemed highly variable, ranging from on demand treatment regimens to intensified dosage maintenance therapies depending on the activity and the severity of the underlying disease.

**Conclusion:** IL-1 inhibitors are a good treatment option for patients with a hereditary recurrent fever syndrome, but the individual need of the dosage of IL-1 inhibitors to control the disease effectively seems highly variable. Severity, activity but also the type of the underlying disease, belong to the parameters underpinning the treat-to-target strategy implemented in an everyday life practice.

## Introduction

Interleukin (IL)-1 is implicated in the pathogenesis of several systemic auto-inflammatory disorders and this recognition has favored the employment of targeted therapies modulating IL-1 signaling in a wide number of diseases (Cavalli and Dinarello, 2015). Several IL-1 inhibitors have been developed, but in France the marketing authorization has been obtained only for two of them, the IL-1 receptor antagonist analog anakinra and the IL-1β selective monoclonal antibody canakinumab. The first one was formerly licensed for rheumatoid arthritis, then cryopyrin-associated periodic syndrome (CAPS), and recently in systemic JIA. The second has an indication in the treatment of systemic JIA and in four hereditary systemic auto-inflammatory disorders (European Medicines Agency, 2018b). In 2018, the pivotal placebo-controlled umbrella study with canakinumab has provided the highest level of evidence for the use of IL-1 blockers to control inflammatory symptoms in 3 diseases other than CAPS: i.e. mevalonate kinase deficiency (MKD), TNF receptor associated periodic syndrome (TRAPS), and familial Mediterranean fever (FMF) (De Benedetti et al., 2018). Anakinra will shortly be licensed in France also for colchicine resistant FMF (crFMF) patients (European Medecines Agency, 2018a).

Despite the studies giving short or medium-term results, the use of IL-1 inhibitors on a long term and especially in real life may differ in terms of both intervals between the injections and dosage. Indeed, patients responding insufficiently to IL-1 inhibition, respond completely to a dose increase or shortening of the interval between the doses (Bodar et al., 2011; Grimwood et al., 2015; Kone-Paut et al., 2017; Deshayes et al., 2018). Conversely, the minimum doses required to treat a patient effectively are less well known, considering that the majority of patients are currently treated with a treat-to-target strategy.

In French tertiary care centers, IL-1 inhibitors have been used off-labeled in theses indications for several years (Meinzer et al., 2011; Stankovic et al.,2012; Rossi-Semerano et al., 2015; Abbara et al., 2017). The analysis of these patients therefore presents an unique opportunity to compare the actual doses received by patients in a nation-wide “real-life” setting to the drug dosage recommended in the product recommendations.

## Materials and Methods

### Study Design and Participants

Patients were identified from the JIR cohort, an international multicenter data repository granted by the Swiss-Children-Rheumatisms foundation, which aims to collect both retrospective and prospective information in a variety of juvenile onset systemic inflammatory disorders (http://www.fondationres.org/fr/jircohorte - NTC02377245). For the purpose of the study, only patients from French centers (pediatric and adult) with complete history data and at least one completed follow-up visit were analyzed. Inclusion criteria to the study were all patients.

1) with a monogenic autoinflammatory recurrent fever syndrome (CAPS, TRAPS, FMF and MKD) according to the EUROFEVER/Printo classification criteria (Gattorno et al., 2019).
2) who received during their follow-up at least one IL-1 inhibitor.

Export of patient’s data took place on 12th June 2017, one month before the marketing authorization of canakinumab in France.

### Protocol Approvals

This study conformed to the tenets of the Declaration of Helsinki and the protocol was approved by the French Ethic Committee (CCTIRS). Patients were enrolled after comprehensive information checking that they (or their legal guardian) were not opposed to the study and the storage of their personal data. The electronic case report form has been the object of an approval of the national commission for Data Protection and Liberties (CNIL).

### Aims and Endpoints

The primary objective of the study was to evaluate the consistence of dosing of IL1inhibitors in HRFs based on European Medicines Agency labeled recommendations.

The secondary aims were 1) to analyze the reasons for discrepancies with the product recommendations and 2) to assess the overall safety profile of IL-1 inhibitors in HRFs.

#### Assessment of the Accordance of the Received Dosage of Medication with the Recommended Dosing Regimen

All the patients who received at least one IL1 inhibitor for colchicine resistant FMF, MKD, TRAPS and CAPS were assessed. Starting and ending date of the IL-1 inhibition were notified so that total exposure time for each IL-1 inhibitor, expressed in patient-years, could be calculated.

To study the different dosage regimens, we considered the dosage of IL-1 inhibitor received at the last visit (or at the last visit before discontinuation of the studied IL-1 inhibitor). Patients were classified into three groups: group 1/lower than recommended dosage, group 2/standard dosage and group 3/intensified dosage. For anakinra, standard dose was defined as 100 mg/day (among adults) or 2 (±0.5) mg/kg/day (among children) (European Medicines Agency, 2018a). For canakinumab the standard dose depended on the indication: for CAPS-patients the standard dose was defined as 150 mg (or 2 (±0.5) mg/kg) every 8 weeks, whereas the standard dose for crFMF, MKD and TRAPS patient was the dose recommended by the European Medicines Agency: 150 mg (or 2 (±0.5) mg/kg) every 4 weeks (European Medicines Agency, 2018b). Patients treated with lower or less frequent injections were considered as receiving lower than recommended doses, whereas those receiving higher dosages or more frequent injections were considered as receiving intensified dosages of canakinumab.

#### Analysis of the Reasons for Discrepancies with the Product Recommendations

To analyze the reasons for accordance or discrepancies of the different dosage regimens with the product recommendations, a descriptive analysis of the treatment modalities of the patients treated with IL-1 inhibitors was performed.

#### Assessment of the Overall Safety Profile of IL-1 Inhibitors

Frequency and description of adverse events were retrieved according to the medDRA terminology. For each adverse event, investigators had to indicate the intensity among “no effect”, “mild”, “moderate”, “severe” and “very severe,” the seriousness with the necessity of an hospitalization or not, the relationship between the medication and the event among “not related,” “not likely,” “possible,” “probable,” and “definitely” and the consequence on the administration of the treatment among “no action,” “drug interrupted,” “drug discontinued,” “dose reduced”. Adverse events were expressed both as absolute number of events during the whole follow-up and as number of events/100 patients/year.

## Results

Forty-five French patients who received at least once an IL-1 inhibitor, either anakinra or canakinumab or both, were identified in the JIR cohort and included for analysis. Table 1 summarizes patient’s characteristics with their treatments. Anakinra was the most given treatment (25/45 – 56%), especially in FMF (9/13 – 69%) and TRAPS (8/8 – 100%) patients. The total treatment exposure to anakinra and canakinumab represented 54 and 202.9 patient-years respectively.

**TABLE 1:** Patients characteristics and received IL1inhibitor.

Disease/Patient *N*°	Mutation (HGVS name)	First line IL1 inhibitor			Second line IL1 inhibitor			Third line IL1 inhibitor	
		Drug	Dosing group	Medication stopped? Y/N Reason	Drug	Dosing group	Medication stopped? Y/N Reason	Drug	Dosing group
CAPS 1	T348M (*p.Thr348Met*)	CAN	Std	Y Patient’s choice					
CAPS 2	D303N (*p.Asp303Asn*)	ANA	Std	Y Scheduled switch from ANA to CAN	CAN	Int	N		
CAPS 3	Y859C (*p.Tyr859Cys*)	CAN	Int	N					
CAPS 4	R260W (*p.Arg260Trp*)	CAN	Std	Y Adverse event (infection)	CAN	Std	N		
CAPS 5	R260W (*p.Arg260Trp*)	CAN	Int	Y Adverse event (metabolic disorder)	CAN	Int	Y Adverse event (nervous system disorder)	CAN	Int
CAPS 6	R260W (*p.Arg260Trp*)	CAN	Std	N					
CAPS 7	T348M (*p.Thr348Met*)	ANA	Std	Y Scheduled switch from ANA to CAN	CAN	Std	N		
CAPS 8	R260W (*p.Arg260Trp*)	CAN	Std	N					
CAPS 9	A352V (*p.Ala352Val*)	CAN	Std	N					
CAPS 10	R260W (*p.Arg260Trp*)	CAN	Int	N					
CAPS 11	R260W (*p.Arg260Trp*)	ANA	Std	Y Scheduled switch from ANA to CAN	CAN	Int	N		
CAPS 12	T348M (*p.Thr348Met*)	CAN	Std	N					
CAPS 13	R260W (*p.Arg260Trp*)	CAN	Int	N					
CAPS 14	T348M (*p.Thr348Met*)	CAN	Int	N					
CAPS 15	R260W (*p.Arg260Trp*)	CAN	Int	N					
CAPS 16	R260W (*p.Arg260Trp*)	ANA	Std	Y Burden of injections	CAN	Std	YPatient’s choice	CAN	Std
CAPS 17	R260W (*p.Arg260Trp*)	CAN	Std	N					
CAPS 18	R260W (*p.Arg260Trp*)	CAN	Std	N					
TRAPS 1	C29S (*p.Cys58Ser*)	ANA	Std	Y Scheduled switch from ANA to CAN	CAN	Low	N		
TRAPS 2	C70Y (*p.Cys99Tyr*)	ANA	Low	YNot effective (on demand)	CAN	Low	N		
TRAPS 3	D42E (*p.Asp71Glu*)	ANA	Low	N					
TRAPS 4	Y20C (*p.Thy49Cys*)	ANA	Low	Y Scheduled switch from ANA to CAN	CAN	Low	N		
TRAPS 5	T50M (*p.Thr79Met*)	ANA	Low	N					
TRAPS 6	C43F (*p.Cys72Phe*)	ANA	Low	N					
TRAPS 7	D42E (*p.Asp42Glu*)	ANA	Low	N					
TRAPS 8	R92Q (p.Arg121Gln)	ANA	Std	Y Not effective (on demand)	CAN	Std	Y not effective	Other	
FMF 1	I692*/V726A (*p.Ile692Del/p.Val726Ala*)	ANA	Std	Y Adverse event (skin disorder)	CAN	Low	N		
FMF 2	M694V/M694V (*p.Met694Val/p.Met694Val*)	ANA	Low	Y Adverse event (hepatitis)	CAN	Low	N		
FMF 3	M694V/M694V (*p.Met694Val/p.Met694Val*)	CAN	Low	N					
FMF 4	M694V/WT (*p.Met694Val/WT*)	ANA	Low	Y Not effective	CAN	Std	N		
FMF 5	M694V/M694V (*p.Met694Val/p.Met694Val*)	ANA	Std	Y Remission					
FMF 6	M694V/WT (*p.Met694Val/WT*)	ANA	Low	N					
FMF 7	M694V/M694V (*p.Met694Val/p.Met694Val*)	CAN	Low	N					
FMF 8	M694V/M694V (*p.Met694Ile/p. Met694Ile*)	ANA	Low	N					
FMF 9	M694V/WT (*p.Met694Val/WT*)	CAN	Low	N					
FMF 10	M694V/WT (*p.Met694Val/WT*)	ANA	Low	N					
FMF 11	M694V/M694V (*p.Met694Val/p.Met694Val*)	ANA	Std	Y Burden of injections	CAN	Low	Y Remission	CAN	Low
FMF 12	M694V/M694V (*p.Met694Val/p.Met694Val*)	CAN	Low	Y Adverse event (infection)					
FMF 13	M694I/M694I (*p.Met694Ile/p.Met694Ile*)	ANA	Std	Y Not effective	Other				
MKD 1	K13Q/N205D (*p.Lys13Gln/p.Asn205Asp*)	ANA	Std	Y Remission	CAN	Low	N		
MKD 2	D204E/V377I (*p.Asp204Glu/p.Val377Ile*)	ANA	Std	Y Scheduled switch from ANA to CAN	CAN	Low	N		
MKD 3	I268T/V377I (*p.Ile268Thr/p.Val377Ile*)	CAN	Low	N					
MKD 4	G309S/R388X (p.Gly309Ser/p.Arg388*)	CAN	Low	N					
MKD 5	G311R/V377I (*p.Gly311Arg/p.Val377Ile*)	ANA	Std	Y Scheduled switch from ANA to CAN	CAN	Std	N		
MKD 6	L51F/WT (*p.Leu51Phe/WT*)	CAN	Int	N					

Low = group 1: patient receiving lower than recommended dosage.

Std = group 2: patient receiving standard dosage.

Int = group 3: patient receiving an intensified dosage of IL-1 inhibitor.

ANA = anakinra. CAN = canakinumab.

The treatment group of the patients (low, standard or intensified) was defined on the dosage received at the last visit (or at the last visit before discontinuation of the studied IL-1 inhibitor).

**FIGURE 1:**
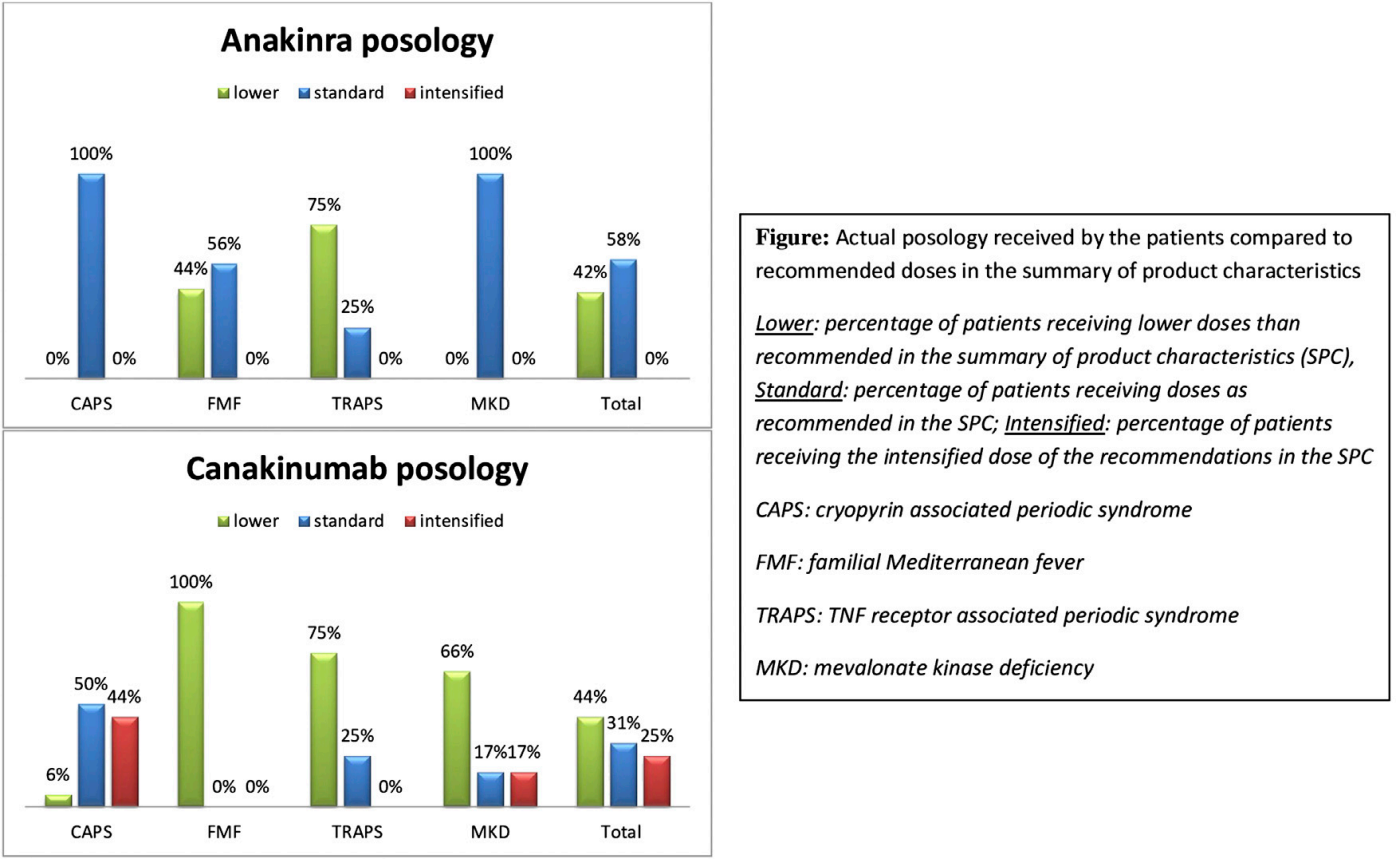
Actual posology received by the patients compared to recommended doses in the summary of product characteristics.

Figure 1 summarizes the actual doses received at the last visit (or at the last visit before discontinuation of the studied IL-1 inhibitor) according to the different diseases. Group 1 (lower dosage than in product recommendations) constituted 43% of the patients, regardless the type of IL-1 inhibitor. This was especially true for FMF, TRAPS and MKD patients on canakinumab with 100%, 75%, and 66% of patients respectively who received less than standard dose (i.e. 150 mg or 2 mg/kg every 4 weeks). Group 2 (standard dose) concerned 42% of the patients; whereas an intensified dose of IL-1 inhibitors (group 3) was given to 15% of the patients: 44% of CAPS patients and 17% of MKD patients received in our cohort higher doses than the recommended standard dose whereas neither FMF nor TRAPS patients required the intensified maintenance dose (i.e. 300 mg or 4 mg/kg every 4 weeks).

The lower dosages in our cohort than the ones recommended in the summary of product characteristics (SPC) were explained by different treatment regimens:

• Fifty percent of the patients (i.e 2 FMF and 3 TRAPS patients) treated with anakinra who received less than the recommended dose were treated with an on-demand regimen (anakinra administration only during flares), the other half received either a maintenance treatment by injections every other day instead of daily injections, or lower daily doses.
• Administration modalities for canakinumab also varied: One CAPS and one FMF patient received an “on-demand” regimen, i.e., an injection of canakinumab only if clinical and biological symptoms appeared. The other lower dose regimens involved patients with the new indication of canakinumab (i.e., FMF, TRAPS and MKD): they received less frequent injections than those stipulated in the SCP, varying from an injection every 10 weeks to every 6 weeks.

**TABLE 2:** Reported side effects with IL1 inhibitors during the study period.

	Anakinra (24 patients 54 pts/year)	Canakinumab (36 patients/202,9 pts/year)
Infections/infestations	7	26
Hepatobiliary disorders	1	1
Metabolism and/or nutrition disorders	0	1
Nervous system disorders	0	3
Skin and/or subcutaneous tissue disorders	3	1
Surgical and/or medical procedures	0	2
Vascular disorders	0	1
Total	11	35

Concerning reported adverse eventsoccuring while on IL1 inhibitors (Table 2), 6 led to a therapeutic discontinuation, whereas 40 other adverse events possibly, probably or certainly related to IL1 inhibition were reported. The global incidence of adverse events with IL1 inhibition was 17.1 per 100 patient/years. No significant difference in the incidence of adverse events was found between anakinra or canakinumab therapy (*p* = 0.55). No link could be established between the frequency of adverse events and the dosage of IL1 inhibitor received. Especially of the nine patients with a side effect considered as serious or very serious by the investigator, three received an intensified dosage regimen. No life-threatening adverse events were retrieved in our study.

**FIGURE 2:**
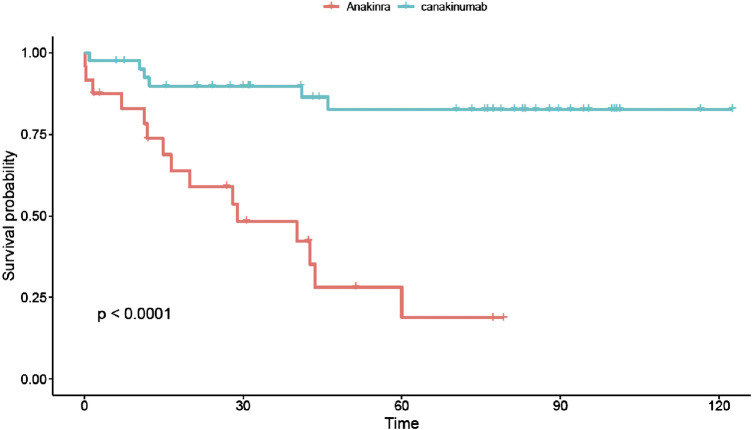
Drug survival curves for anakinra (red line) and canakinumab (blue line). The retention rate for canakinumab was significantly higher than for anakinra. Time expressed in months.

The global drug retention rate was higher for canakinumab than anakinra (Figure 2): 33 out of 36 patients (92%) that ever received canakinumab continued the treatment at the end of the study period, whereas this was only the case for 7 out of 25 (29%) of anakinra treated patients (*p* < 0.0001).

## Discussion

This study assessed the dosing regimen of IL-1 inhibitors in patients with a monogenic auto-inflammatory disease. During the study period, in France licensed use of IL-1 inhibitors was possible only in CAPS patients. Nevertheless the French healthcare organization enables physicians belonging to secondary or tertiary care centers for rare diseases to prescribe off labeled drugs and our study focused on these patients.

Almost half of the patients received lower dosages of IL-1 inhibitors than the recommended standard dose. These lower dosage regimens concerned 60% of the patients with the more recent licensed indications of IL1-inhibitors: crFMF, TRAPS and MKD (De Benedetti et al., 2018; European Medicines Agency, 2018b). Especially, canakinumab injections rate was far lower and varied greatly from one patient to another with injections ranging from every 6 to every 10 weeks. This was probably due to the fact that patients received doses based upon the licensed use of canakinumab (ie CAPS, in whom the standard dose is lower than in the other recurrent autoinflammatory fever syndromes). Indeed the publication of the phase 3 Canakinumab Pivotal Umbrella Study in Three Hereditary Periodic Fevers (CLUSTER) study (De Benedetti et al., 2018), defining the standard dose of 150 mg (or 2 mg/kg) every 4 weeks, occurred after the end of our study. Nevertheless it is a striking finding that in a real-life setting, lower doses than the anticipated standard dose seem sufficient to control the disease. Moreover it seems to show that the need for IL1 inhibitors is not uniform: while 100% of patients with crFMF responded to low doses of interleukin 1 inhibition, patients with MKD required overall higher doses, with a need of intensified doses observed only in this group. TRAPS patients seem to display an intermediate profile of interleukin 1 thresholds, with more various needs of the level of IL-1 inhibition to control the disease. Thus results show that the optimal dosage for properly treating any of these diseases is not yet fully defined.

The other main reason for lower dosages was an on-demand treatment strategy in FMF and TRAPS patients. An on-demand strategy was previously described only in 3 studies with anakinra (Bodar et al., 2011; Grimwood et al., 2015; Babaoglu et al., 2019). In a real life setting, this strategy seems to be a realistic treatment option for selected patients (equally well with anakinra than canakinumab), as 5 out of 7 patients still received an on-demand regimen at the end of the study period. Both patients not responding to an on demand treatment with anakinra switched to a maintenance therapy with canakinumab with – according to the including physician – a good response.

The global incidence rate of adverse events in our study was slightly higher than in an Italian study (17.1 per 100 patient/years in our study vs. 8.4 in the Italian study) (Sota et al., 2018), but only already known side effects were described by the participating physicians (Table 2), with mainly–as anticipated-infectious complications (∼11 per 100 patient/years). Most adverse events were considered to be mild and could be managed with minimal treatment modifications. No death, no neoplasm, no *tuberculosis* infection or reactivation, nor opportunistic infections were reported in our study. Our observations are comforting about the safety profile of IL1 inhibitors in HRFs and support the hypothesis that severe adverse events with IL1 inhibitors are preferentially related to the underlying diseases requiring IL1 inhibition and to the poor general clinical condition, rather than to an actual effect of IL-1 blockade (Sota et al., 2018).

We show a far better drug retention for canakinumab than for anakinra, whereas side effects seemed equally frequent in both groups. Our hypothesis is that the ease of treatment may be the most important point for treatment persistence in patients. It is worth noting, that during the scheduled switch from anakinra to canakinumab, none of the attending physicians pointed out that anakinra was not sufficiently effective to justify changing the medication. Similarly, patients with on-demand anakinra therapy with inadequate disease control switched directly to canakinumab–and not daily anakinra - maintenance therapy. These observations suggest that the ease of treatment is also a major argument guiding the choice of the drug for the prescribing physician.

The major flaw of our study is that due to the retrospective design of our study; we were not able to retrieve a standardized disease activity score and consequently we were not able to link the disease activity of the patients to their treatment regimens. However we consider that we can infer the control of disease activity indirectly by assuming that the adaptations of therapies decided by the investigating physician were made because of criteria related to the severity and the control of the disease. The observed highly variable treatment regimens, ranging from on demand treatment regimens to intensified dosage maintenance therapies, reflects in our opinion that in daily life the investigating physicians adapts drug dosages as closely as possible to disease activity. This is all the more true since our study took place before the French marketing authorization for IL1-inhibitors in HRFs, at a time when dosages had not yet been standardized by the SCP.

A second bias of our study concerns the heterogeneity of our sample, particularly concerning pathologies. However, this heterogeneity also highlighted that individual treatment needs are highly variable. Future studies should focus on identifying and refining the parameters underpinning the treat-to-target strategy practiced in HRFs.

### Key Messages

- (1) IL-1 inhibitors are a good treatment option for patients with a hereditary recurrent fever syndrome.
- (2) The individual need of the dosage of IL-1 inhibitors to control the disease effectively seems highly variable, with about 45% of patients responding well to low dosages of IL-1 inhibitors.
- (3) On-demand treatment with a short half-life IL-1 inhibitor may be a treatment option for some selected patients with a recurrent hereditary fever syndrome.

## Data Availability Statement

The raw data supporting the conclusions of this article will be made available by the authors, without undue reservation.

## Ethics Statement

The studies involving human participants were reviewed and approved by French Ethic Committee (CCTIRS). Patients were enrolled after comprehensive information checking that they (or their legal guardian) were not opposed to the study and the storage of their personal data.

## Author Contributions

VH and SG-L were involved in the conception and design of the study. VH, SG-L, IK-P, AB, CG, GG, AC, AP, MH, and PP organized the data base. VH and MD analyzed the data. VH wrote the first draft of the manuscript. All authors contributed to the manuscript revision, read and approved the submitted version.

## Funding

No specific funding was received from any bodies in the public, commercial or not-for-profit sectors to carry out the work described in this article.

## Conflict of Interest

VH, IK, GG, MH, and SG-L received personal fees and non-financial support from Novartis and SOBI; CG and AB received non-financial support from Novartis; AP received non-financial support from SOBI.

The remaining authors declare that the research was conducted in the absence of any commercial or financial relationships that could be construed as a potential conflict of interest.
